# Optimal precursor ion selection for LC-MALDI MS/MS

**DOI:** 10.1186/1471-2105-14-56

**Published:** 2013-02-18

**Authors:** Alexandra Zerck, Eckhard Nordhoff, Hans Lehrach, Knut Reinert

**Affiliations:** 1 Department of Vertebrate Genomics, Max Planck Institute for Molecular Genetics, Ihnestr. 63-73, Berlin, 14195, Germany; 2, Current address: M2-Automation, Berlin, Germany; 3 Department of Computer Science, Free University Berlin, Takustr. 9, Berlin, 14195, Germany

## Abstract

**Background:**

Liquid chromatography mass spectrometry (LC-MS) maps in shotgun proteomics are often too complex to select every detected peptide signal for fragmentation by tandem mass spectrometry (MS/MS). Standard methods for precursor ion selection, commonly based on data dependent acquisition, select highly abundant peptide signals in each spectrum. However, these approaches produce redundant information and are biased towards high-abundance proteins.

**Results:**

We present two algorithms for inclusion list creation that formulate precursor ion selection as an optimization problem. Given an LC-MS map, the first approach maximizes the number of selected precursors given constraints such as a limited number of acquisitions per RT fraction. Second, we introduce a protein sequence-based inclusion list that can be used to monitor proteins of interest. Given only the protein sequences, we create an inclusion list that optimally covers the whole protein set. Additionally, we propose an iterative precursor ion selection that aims at reducing the redundancy obtained with data dependent LC-MS/MS. We overcome the risk of erroneous assignments by including methods for retention time and proteotypicity predictions. We show that our method identifies a set of proteins requiring fewer precursors than standard approaches. Thus, it is well suited for precursor ion selection in experiments with limited sample amount or analysis time.

**Conclusions:**

We present three approaches to precursor ion selection with LC-MALDI MS/MS. Using a well-defined protein standard and a complex human cell lysate, we demonstrate that our methods outperform standard approaches. Our algorithms are implemented as part of OpenMS and are available under http://www.openms.de.

## Background

LC-MS/MS-based proteomics is a key technique for protein quantitation and identification. A typical workflow starts with the proteolytic digestion of protein samples, using usually trypsin. The resulting peptide mixture is inserted into a liquid chromatography (LC) column in which the peptides are eluted at different time points, called retention time (RT), according to their physicochemical properties (e.g. hydrophobicity and polarity). LC system and mass spectrometer are connected, either directly with Electrospray-MS (ESI-MS) or indirectly via fractionation onto a target plate as used in MALDI-MS. The resulting peptide signals in the LC-MS map are referred to as *features* while the selection of features for fragmentation with MS/MS is called *precursor ion selection*. Peptide identifications are assigned to MS/MS spectra using database search tools, such as Mascot [1] or X!Tandem [2], or by *de novo* sequencing [3,4]. The peptide sequences are then used to reconstruct the proteins that were present in the sample.

A problem for protein identification with tandem mass spectrometry is the limited number of possible MS/MS acquisitions. Even in simple protein digests there are more detected peptide signals than possible selections for MS/MS [5]. The number of possible fragmentations is either limited by the elution time of the peptide (ESI) or by the amount of sample available for each fraction (MALDI). A standard method for precursor ion selection with ESI-MS/MS is data dependent acquisition (DDA) which selects the *x* most intense signals in each MS spectrum for fragmentation, with *x* depending on the instrument type. However, as biological samples have a high dynamic range of protein abundance, the number of peptide identifications is biased towards high-abundance proteins, although low-abundance proteins are mostly of higher interest.

In order to circumvent redundancy, DDA can be combined with a dynamic exclusion list (DEX) that prevents fragmenting a signal at the same *m* / *z*-value within a specified RT range. Exclusion lists are often used for replicate analyses [6-11]: after each LC-MS/MS run the exclusion list is updated and contains the fragmented or identified signals of previous runs. In comparison to simple repetitions, Chen et al. [7] showed that the number of unique peptide identifications can be significantly increased. Bendall et al. [10] reached a higher number of proteins identifications.

A complementary strategy to exclusion lists is directed MS/MS. Instead of excluding potentially uninteresting signals, the selection focusses particularily on signals of interest. These signals are part of an inclusion list that contains the *m* / *z*-values and usually an RT window for each peptide. This procedure is a typical approach for LC-MALDI MS/MS where MS and MS/MS are decoupled. Thus, MS acquisition can be used to create a map of all detected signals which guides the precursor ion selection. Moreover, inclusion lists have also been used in combination with ESI-MS/MS: a consensus map of detectable LC-MS features created from previous runs was used to create the inclusion list [12-15]. These studies showed that compared with DDA directed MS/MS might identify a higher number of peptides [14,15]. This effect is more pronounced for low intensity peptides [14].

In the last years, in several studies iterative approaches for precursor ion selection were applied. For instance, Scherl et al. [16] added theoretical *m* / *z*-values of tryptic peptides of already identified proteins to an exclusion list. In a previous study, we showed the effect of combining both the directed analysis of interesting signals and the exclusion of uninteresting signals through a heuristic [17]. In our study, a prioritized list of all possible precursors was reranked during ongoing MS/MS acquisition based on the identifications yielded so far. Precursors having an *m* / *z*- value matching tryptic peptides of already identified proteins received a lower priority, whereas precursors matching tryptic peptides of uncertain protein candidates were assigned higher priorities. We demonstrated that this strategy can identify the same number of proteins as standard methods using fewer precursors. In our study, theoretical peptides were matched onto observed features using only the *m*/*z*-value. Thus, our method showed a clear dependence on mass accuracy and sample complexity.

Liu et al. [18] developed an iterative MS/MS acquisition (IMMA) approach that used different filtering techniques to exclude uninteresting signals. Proteotypic peptides of already identified proteins are excluded as well as signals with a mass defect untypical for peptides. This way, a larger number of proteins could be identified than with DDA.

In this manuscript, we introduce a deterministic framework that formulates the precursor ion selection problem as Integer Linear Program (ILP). We show that it can be easily adapted to variations of the original problem. We present three different scenarios and their corresponding optimization problems. We address the problem of erroneous peptide-precursor assignments by including predictions of RTs and proteotypic peptides into the matching. Furthermore, we employ a probabilistic scoring to infer proteins from peptide identifications. Our methods are implemented as part of the open-source library OpenMS [19] and will be available as a TOPP tool [20] as part of the next release of OpenMS.

## Methods

Several precursor ion selection strategies are conceivable depending on the aim of a study and the available prior knowledge about the sample. Here, we focus on three settings: the first two use static inclusion lists created once before the MS/MS acquisition starts. The third changes the selection based on previous identifications. The two static inclusion list approaches differ in the information used during the selection process. In the following, when talking about peptides we refer to protein subsequences as opposed to precursors which denote MS/MS measurements. 

• **Feature-based inclusion list**: Given an LC-MS feature map, we want to maximize the number of scheduled precursors given some constraints on the number of simultaneous acquisitions per RT fraction. This is a common scenario with LC-MALDI due to its decoupled nature of LC and MS.

• **Protein-based inclusion list**: Given a list of protein sequences but no prior LC-MS run, we want to find an optimal set of precursors that represents the proteins of interest best. As proteins are not identified directly we need to find a peptide set that optimally covers our specific proteins. For this peptide set, we predict the LC-MS features (i.e. retention time and *m* / *z* acquisition window) and add them to the inclusion list.

• **Iterative precursor ion selection**: Given an LC-MALDI-MS feature map, we want to optimally exploit the set of possible precursors. Optimality in this case means that we want to identify the proteins in a sample using a minimal set of precursors, so that the remaining precursors can be used to discover other proteins. The precursor ion selection shall be adjusted during the ongoing MS/MS acquisition based on previous peptide and protein identifications. This way, we combine the discovery nature of DDA with directed MS/MS. As with both inclusion list formulations, the number of MS/MS acquisitions is limited by the number of precursors per RT fraction.

These settings can be formulated as optimization problems, which can be formalized as Integer Linear Programs (ILP). Solving the ILPs yields a list of precursor ions, the actual inclusion list. In the following sections we will introduce and explain the formulations.

### Feature-based inclusion list

**Figure 1:**
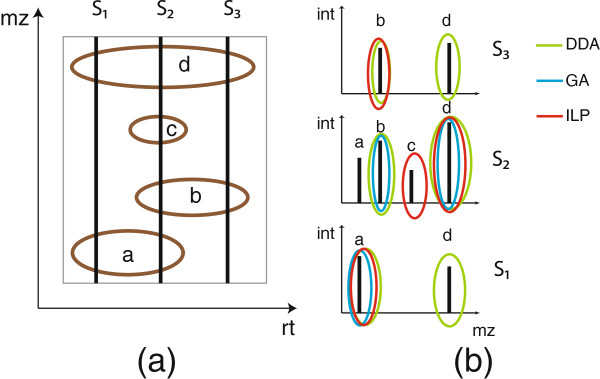
**Feature-based precursor ion selection.** (**a**) LC-MS map containing four features *a* - *d*. (**b**) MS spectra of the same map with feature peaks. The coloured ellipses show the selected precursors for the different strategies: green with DDA, blue with GA and red with the ILP. Limiting the number of precursors to 2 per spectrum, feature *c* is never chosen neither with DDA nor with GA. With GA no feature is selected in spectrum *S*_3_. The ILP selection selects all four features once.

Given a feature map, we want to schedule the highest possible number of features as precursors for MS/MS-fragmentation. Since a feature elutes over several scans we have the option to choose the feature as precursor in any of those scans. Ideally, one would like to use for each feature a fraction with a high signal intensity for fragmentation. A greedy approach (GA) chooses for each feature the fraction with the highest signal intensity. Then, in each fraction the highest of these feature maxima are scheduled for MS/MS. However, situations can be constructed where GA selects less features than a global strategy that tries to optimize the selection for all features simultaneously. An illustration of such a situation is shown in Figure 1. In the following, we present a formulation of the feature-based precursor ion selection as optimization problem.

For each feature *j*, we introduce a set of binary variables *x*_*j*,*s*_, which are set to 1 if we choose feature *j* in scan *s* as a precursor and 0 otherwise. Since we want to choose the best possible fraction for each precursor, we do not simply maximize the number of scheduled precursors but use the feature intensities as weights, because high intensity features are more likely to produce good and interpretable MS/MS spectra. The intensities are normalized by the maximal signal intensity the respective feature has in any spectrum. This results in weights between 0 and 1 and prevents a bias towards selecting only high intensity features.

We have two constraints: first, the capacity of spectrum *s*, i.e., the maximal possible number of acquired MS/MS spectra in spectrum *s*. And second, the number of times a feature can be selected as a precursor which is set to 1 here, but this constraint could easily be relaxed to other values. Table 1 gives an overview of all variables and constants used in the LP formulations.

**Table 1:** Variables and constants used in LP formulations

**Variable name**	**Explanation**
*x*_*j*,*s*_	Indicator variable, 1 if feature *j* is selected in spectrum *s*,
	0 otherwise
*x*_*j*_	Indicator variable, 1 if feature *j* is part of the solution,
	0 otherwise
*int*_*j*,*s*_	Normalized signal intensity of feature *j* in spectrum *s*
*cap*_*s*_	Maximal number of MS/MS precursors in spectrum *s*
*D*_*i*_	Detectability of protein *i*
*y*_*i*_	-*log*(1-*D*_*i*_), higher values reflect a better protein detectability
*d*_*k*_	Detectability of peptide *k*
*a*_*i*,*k*_	Indicator variable, 1 if peptide *k* is part of protein *i*,
	0 otherwise
*ws*	RT window size
*t*_*p*_	Predicted RT
*max*_*list*_*size*	maximal number of elements in inclusion list
*p*_*k*_	Probability that peptide *k* was identified correctly
*P*_*i*_	Probability that protein *i* was identified correctly
*k*_1_,*k*_2_,*k*_3_	Weights
*b*_*i*_	Indicator variable, 1 if the protein probability of protein *i* is at least *c*, 0 otherwise
*c*	Minimal protein probability to declare a protein identified
*z*_*i*_	*z*_*i*_ = 1 if *P*_*i*_ ≥ *c*, otherwise *z*_*i*_ ∈ [0, 1)
*M*_*k*_	Set of features having an *m*/*z* within a specified ppm range around the theoretical *m*/*z* of peptide *k*
*m*_*k*,*j*_	Matching probability of feature *j* with peptide *k*
*precs*	Number of already fragmented precursors
*step*_*size*	Number of selected precursors in each iteration

The LP formulation of the feature-based inclusion list can be formalized in the following way:

(1) $$\begin{array}{ll} \max\underset{j,s}{\sum }x_{j,s}\cdot & \text{in}t_{j,s}\; \end{array}$$

(2) $$\begin{array}{ll} \text{subject to}\;\forall_{s}:\underset{j}{\sum }x_{j,s} & \leq \text{ca}p_{s}\; \end{array}$$

(3) $$\begin{array}{ll} \forall_{j}:\underset{s}{\sum }x_{j,s} & \leq 1\; \end{array}$$

(4) $$\begin{array}{ll} x_{j,s} & \in \{0,1\}.\; \end{array}$$

Here, *x*_*j*,*s*_ is an indicator variable, it is 1 if feature *j* is selected in spectrum *s* and 0 otherwise. *int*_*j*,*s*_ is the normalized intensity of feature *j* in spectrum *s*, and *cap*_*s*_ is the “capacity” of spectrum *s*, i.e., the maximal possible number of precursors for that spectrum. The problem of finding an optimal inclusion list is an instance of a well-known combinatorial problem, the Knapsack problem. We will show that solving our ILP yields a global optimal inclusion list.

In our implementation, we solve the ILP formulation using the GNU Linear Programming Kit (GLPK, http://www.gnu.org/software/glpk/). The solution provides values for all *x*_*j*,*s*_ and all features *j* where *x*_*j*,*s*_ = 1 are part of the final inclusion list. Due to Constraint (3), *x*_*j*,*s*_ can only be 1 for at most one *s* for each precursor *j*. Thus, each precursor is scheduled in a specified fraction.

### Protein sequence-based inclusion list

**Figure 2:**
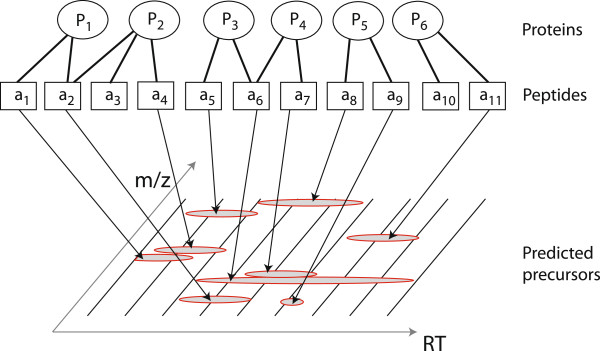
**Protein sequence-based inclusion list.** Given a set of proteins of interest *P*_1_ to *P*_6_, we compute all their possible tryptic peptides *a*_1_ to *a*_11_. For each of the peptides, we calculate their *m* / *z*, and predict the RT and their likelyhood to be detectable in a given experimental setup. With these values we create a map of predicted features that are used for the inclusion list.

In the last section, we developed a method for inclusion list creation based on LC-MS feature maps. In the following, we describe another inclusion list scenario: protein-based precursor ion selection. Given a list of protein sequences that we want to identify in a sample, the task is to select a set of precursors that represents the whole set of proteins. However, with LC-MS/MS we cannot identify proteins directly. Instead, we need to collect peptide identifications that can afterwards be assembled to protein identifications. As it is known which peptide sequences are part of which protein sequence, we can select a set of peptides that yields a sufficient protein coverage. For these peptides we can predict the RT and calculate their *m* / *z*-value. This way, we retrieve a set of possible precursors based on a set of protein sequences. Figure 2 shows the relation between precursors, peptides and proteins.

This problem can be formulated as an optimization problem as well. Again, we have the spectrum capacity and the number of times a feature can be selected as constraints. Additionally, we want to achieve a certain likelihood for each protein to be identified with the selected precursors. In the following, we will refer to this as the protein detectability, in analogy to peptide detectability which is the likelihood to detect and identify a peptide in a given experimental setup. We develop a formula to compute the protein detectability via the protein probability calculation in the next section. This finally leads to the formulation of the protein sequence-based LP.

#### Protein probability calculation

A basic problem for shotgun proteomics is inferring protein identifications from peptide identifications. Several approaches for the protein inference problem were published during the last years [21-23]. In order to calculate protein probabilities, we use the basic formulas of ProteinProphet [22], a widely used tool part of the Trans-ProteomicsPipeline [24]. Thus, the probability *P*_*i*_ that protein *i* is present in the sample can be calculated as the probability that at least one of its constituting peptides was correctly identified:

(5) $$P_{i}=1-\underset{j}{\prod }(1-p_{j})\;.$$

Here, *p*_*j*_ is the probability that peptide *j* is correctly identified. As peptide *j* might be part of more than one protein, ProteinProphet uses additional weights to distribute the contribution of *p*_*j*_ to several proteins. Using the logarithm, we reformulate the product in Equation (5) to a summation:

(6) $$\begin{array}{ll} \underset{j}{\prod }(1-p_{j})=1-P_{i} & \; \end{array}$$

(7) $$\begin{array}{ll} \underset{j}{\sum }\text{log}(1-p_{j})=\text{log}(1-P_{i}), & \; \end{array}$$

which is invalid for *P*_*j*_ = 1, so in this case we enter a pseudo count instead.

#### Prediction of peptide properties

In shotgun LC-MS/MS experiments typically not all tryptic peptides of a protein are observed. Instead, a characteristic set of peptides exists which can be often identified for a specific protein, these peptides are called proteotypic peptides [25]. Closely related to proteotypicity the detectability of a peptide is the likelihood that the molecular ions of the peptides are detected, fragmented by MS/MS and identified through a database search. There exist several approaches to predict peptide detectabilities [26-29]. We use a machine-learning approach from Schulz-Trieglaff et al. [29] for the prediction of the detectability of a peptide and denote it with *d*_*p*_. As *d*_*p*_ is a likelihood, it ranges between 0 and 1.

Similar to detectability prediction it is also possible to predict the retention time of a peptide. Again, we use a machine-learning approach to predict the RTs of peptides [30]. In our approach, we then assume an RT window around the predicted RT for the precursor ion selection.

Both methods for detectability and RT prediction use support vector regression (SVR) with a kernel function that works solely on the peptide sequence. For RT prediction, a training set of peptide identifications with accurate retention times is required. Model training for detectability prediction requires a positive set of observed peptides and a set of undetectable peptides.

#### >Calculation of protein detectabilities

When creating the protein sequence-based ILP, there are no MS/MS measurements available. Therefore, peptide probabilities can not be considered. Instead, we substitute them by peptide detectability which reflects the likelihood of a peptide to be detected and identified by MS/MS. We calculate the detectability of protein *i* (*D*_*i*_) via:

(8) $$\begin{array}{ll} \underset{p,\text{with}a_{\text{ip}}=1}{\sum }\underset{s}{\sum }\text{log}(1-d_{p}\cdot x_{p,s})=\text{log}(1-D_{i}), & \; \end{array}$$

where *a*_*ip*_ is 1 if peptide *p* is part of protein *i* and 0 otherwise. *x*_*p*,*s*_ indicates whether a peptide *p* is part of the inclusion list and selected as precursor in spectrum *s*. In our specific case the indicator variable *x*_*p*,*s*_ can only be 0 or 1, hence the left hand side sum in Equation (8) equals:

(9) $$\begin{array}{ll} \underset{p,\text{with}a_{\text{ip}}=1}{\sum }\underset{s}{\sum }x_{p,s}\cdot \text{log}(1-d_{p})=\text{log}(1-D_{i}). & \; \end{array}$$

#### LP formulation

In the following, we use the previously developed protein detectabilities for the creation of inclusion lists to maximize the sum of the protein detectabilities.

(10) $$\begin{array}{cccc} & \; & \max\sum y_{i} & \; \end{array}$$

(11) $$\begin{array}{cccc} \text{s. t.}\;\; & \forall_{s}:\; & \underset{j}{\sum }x_{j,s} & \leq \text{ca}p_{s}\; \end{array}$$

(12) $$\begin{array}{cccc} & \forall_{j,s}:\; & x_{j,s} & \leq x_{j}\; \end{array}$$

(13) $$\begin{array}{cccc} & \; & \underset{j}{\sum }x_{j} & \leq \text{max}\text{\_}\text{list}\text{\_}\text{size}\; \end{array}$$

(14) $$\begin{array}{cccc} & \forall_{j}:\; & \underset{s\notin [t_{p-\text{ws}},t_{p+\text{ws}}]}{\sum }x_{j,s} & =0\; \end{array}$$

(15) $$\begin{array}{cccc} & \forall_{i}:\; & y_{i} & =-\text{log}(1-D_{i})\; \end{array}$$

(16) $$\begin{array}{cccc} & \forall_{j,s}:\; & x_{j,s},x_{j} & \in \{0,1\}\; \end{array}$$

*t*_*p*_ denotes the predicted RT for peptide *p* and *ws* is the RT window size.

Again, solving the LP formulation using a solver like GLPK yields a set of variables *x*_*j*,*s*_ = 1 that build the inclusion list. In this case, we provide RT windows for each precursor in the inclusion list. Thus, for each precursor *j* there can be multiple *x*_*j*,*s*_ = 1.

### LP for iterative precursor ion selection

The methods described in the previous sections are used for inclusion list creation prior to MS/MS acquisition. In the following, we develop an LP formulation for iterative precursor ion selection where the selection is adapted during ongoing MS/MS acquisition. In contrast to replicate analyses, where new LC-MS and LC-MS/MS measurements are performed in each replication step, in iterative MS/MS acquisition the same sample and the same LC-MS map is used for the whole analysis. This is especially suited for LC-MALDI MS/MS as there the sample is “frozen” on the target and data acquisition can be suspended. After the initial LC-MS step an LC-MS feature map is created for the sample which is used for precursor ion selection. During the iterative analysis, in each iteration a set of precursors is chosen whose MS/MS acquisition is triggered. Variables corresponding to the selected precursor set are fixed for subsequent iterations. As we describe methods for LC-MALDI, we can step forward and backward “in time” by selecting fractions corresponding to different, not necessarily consecutive RTs.

The goal of the iterative precursor ion selection is twofold. On the one hand, a maximal number of proteins shall be identified with a given statistical confidence. On the other hand, a maximal possible number of precursors shall be fragmented which is limited by the available sample. For both aims, LP formulations were presented in the last sections. For the iterative precursor ion selection we combined these LPs. After each iteration, a database search is performed for each MS/MS spectrum. Afterwards, the LP formulation is adapted based on the search results. In the following, we describe the iterative workflow in detail.

**Figure 3:**
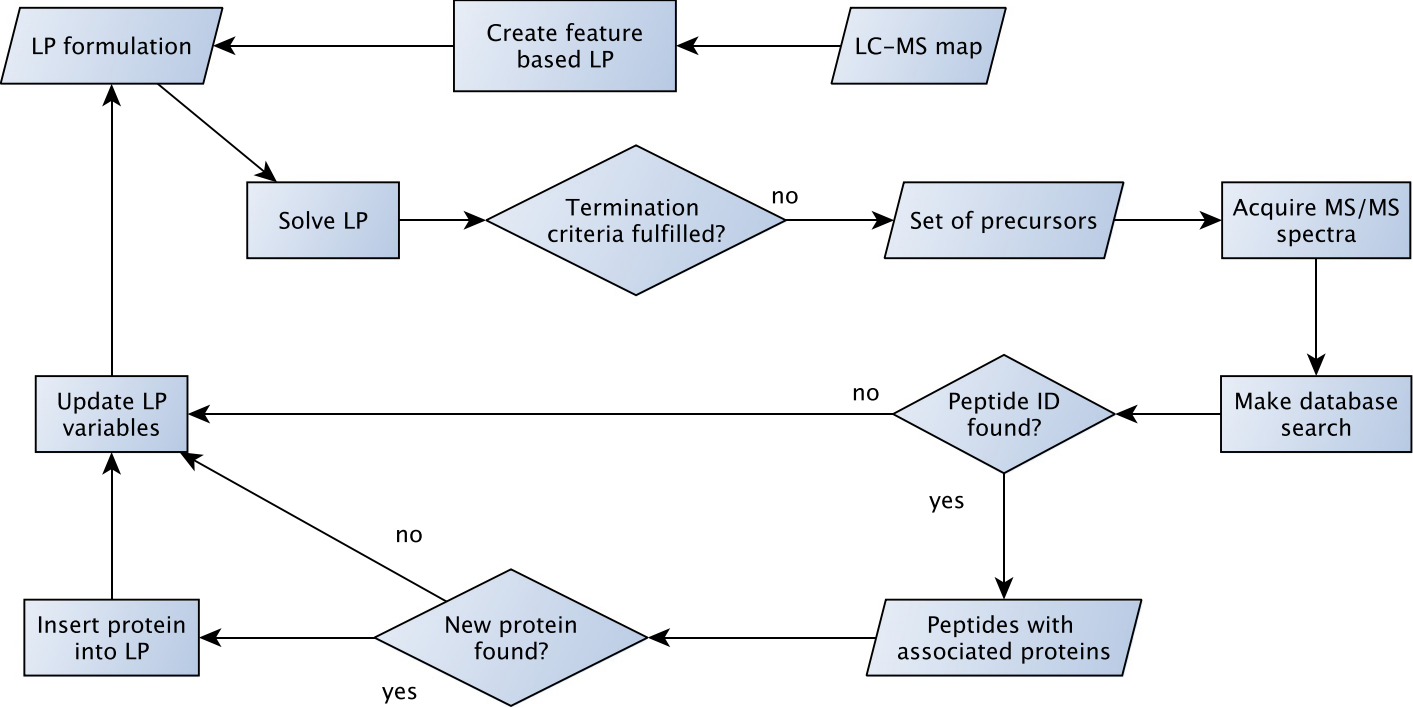
**Workflow of iterative precursor ion selection.** Starting from an LC-MS map, the iterative precursor ion selection creates a feature-based LP and solves it. This way, a set of precursors is selected for which MS/MS acquisition is triggered. After a database search new protein hits are inserted into the LP formulation and all LP variables are updated. Afterwards, the LP is solved again, leading to a new set of selected precursors.

As shown in Figure 3, we start with the LC-MS map based on which a feature-based LP is created. By solving the LP we retrieve a set of precursors for which the fragmentation is triggered. Subsequently, we perform a database search for the resulting MS/MS spectra and assign peptide probabilities to the peptide hits. Then, variables and constraints of the LP formulation are updated: if a new protein was found it is inserted into the LP. Additionally, variables of already fragmented precursors are fixed and peptide weights updated. Afterwards, the LP is solved again yielding a new set of precursors. This procedure is iterated until a predefined termination criterion is fulfilled. This can either be a maximal number of iterations or selected precursors, or the drop of the efficiency of the last *x* spectra below a given threshold. Note, efficiency is given by the number of identified proteins per iteration. Additionally, the iterative precursor ion selection terminates if there exist no LC-MS features that contribute positively to the objective function.

Considering proteins in the LP has two main advantages: first, we want to target peptides hitting protein candidates. These are proteins for which we received peptide identifications, but that did not reach a sufficient significance to declare a protein identified. That way, lower intensity features are included into the precursor set which are likely to yield the missing identifications. On the other hand, signals potentially derived from already identified proteins contribute less weight to the objective function as these do not provide additional information.

Protein probabilities *P*_*i*_ are computed as explained in the context of the protein sequence-based ILP. For the iterative precursor ion selection we require a minimal protein probability *c* to declare a protein identified. Thus,

(17) $$\begin{array}{ll} P_{i} & \geq c\; \end{array}$$

(18) $$\begin{array}{ll} \Rightarrow \text{log}(1-P_{i}) & \leq \text{log}(1-c)\; \end{array}$$

(19) $$\begin{array}{ll} \;\Leftrightarrow \;\frac{\text{log}(1-P_{i})}{\text{log}(1-c)} & \geq 1.\; \end{array}$$

This way, we can define the indicator *b*_*i*_ which is 1 if *P*_*i*_ ≥ *c* and 0 otherwise:

(20) $$b_{i}=\left\lfloor \frac{\text{log}(1-P_{i})}{\text{log}(1-c)}\right\rfloor .$$

#### *Matching of predicted peptides to observed LC-MS features*

Every time we find a new protein hit, we consider all its tryptic peptides and determine their matching LC-MS features. Therefore, a feature set *M*_*p*_ is defined for each peptide *p*, containing all features within a predefined *m* / *z*-range around the theoretical *m* / *z* of *p*. Then, peptide detectabilities and peptide RTs are used to compute matching probabilities. *m* / *z*-values are only used to create a set of matching features – those within a specified *m*/*z*-range of peptide *p* – for which probabilities are computed. *m* / *z* - *values* and mass accuracy are themselves not included in the actual matching probability.

**Figure 4:**
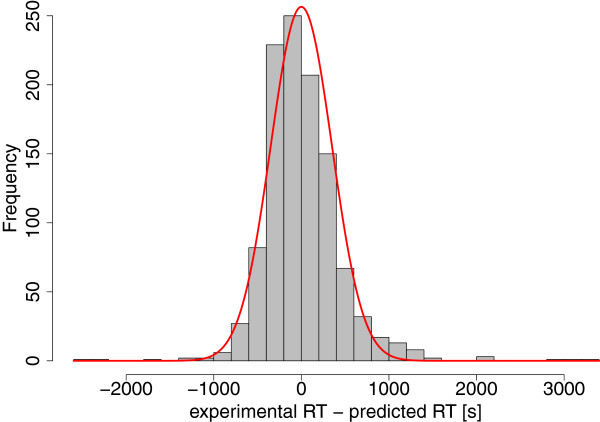
**RT prediction.** RT prediction error for HEK293 with Gaussian distribution in red.

**Figure 5:**
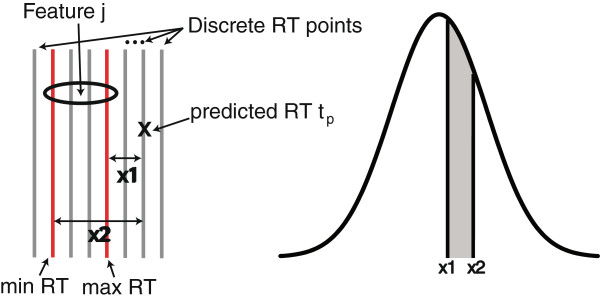
**RT matching probability.** The RT prediction error presumably follows a Gaussian distribution. Thus, the probability that the predicted RT *t*_*p*_ results in an observed feature *j* corresponds to the grey area under the curve and can be computed with the cumulative Gauss function.

We assume the RT prediction error to follow a Gaussian distribution, which is supported by Figure 4. Then, we compute the RT matching probability of a feature *j* and a peptide *p* as the probability that the prediction error is in the range of [*x*_1_,*x*_2_], with *x*_2_ = *t*_*p*_ - *minRT*(*j*) and *x*_1_ = *t*_*p*_ - *maxRT*(*j*). See Figure 5 for an illustration. The probability that a predicted RT *t*_*p*_ is truly shifted by *x* spectra can be calculated as:

(21) $$P(t_{p}-t_{\text{obs}}=x)=\frac{1}{\sigma \sqrt{2\Pi }}\cdot e^{-\frac{1}{2}{\left(\frac{t_{p}-x-\mu }{\sigma }\right)}^{2}}.$$

Considering the minimal and maximal RT of the observed feature *j* leads to an RT matching probability *r*_*p*,*j*_ computed as:

(22) $$\begin{array}{ll} r_{p,j} & =P(x_{1}\leq t_{p}-t_{\text{obs}}\leq x_{2})\\ & =P(t_{p}-t_{\text{obs}}\leq x_{2})-P(t_{p}-t_{\text{obs}}\leq x_{1}). \end{array}$$

Peptide detectabilities and RT matching probabilities are considered to be independent, thus, both are multiplied to determine the probability that feature *j* is produced by peptide *p*:

(23) $$m_{p,j}=d_{p}\cdot r_{p,j}.$$

Now, we combine everything and receive the following LP formulation:

(24) $$\;\begin{array}{cc} \max\;\;\;\; & \overset{\text{protein-based inclusion}}{\overset{︷}{k_{1}\underset{i}{\sum }z_{i}}}\;\;+\overset{\text{feature-based inclusion}}{\overset{︷}{k_{2}\underset{j,s}{\sum }x_{j,s}\cdot \text{in}t_{j,s}}}\\ \;\; & -\overset{\text{exclusion}}{\overset{︷}{k_{3}\underset{i}{\sum }b_{i}\cdot \underset{p}{\sum }\underset{j\in M_{p}}{\sum }\underset{s}{\sum }a_{i,p}\cdot m_{p,j}\cdot x_{j,s}}} \end{array}$$

(25) $$\;\begin{array}{cc} \text{s. t.}\;\;\;\forall_{i}:\;z_{i} & \leq \frac{\text{log}(1-P_{i})}{\text{log}(1-c)}\\ +\frac{\underset{p}{\sum }\underset{j\in M_{p}}{\sum }\underset{s}{\sum }x_{j,s}\cdot \text{log}(1-a_{i,p}\cdot m_{p,j})}{\text{log}(1-c)} \end{array}$$

(26) $$\begin{array}{lll} & \forall_{i}:\; & z_{i}\in [0,1]\; \end{array}$$

(27) $$\begin{array}{lll} & \forall_{s}:\; & \;\;\;\;\underset{j}{\sum }x_{j,s}\leq \text{ca}p_{s}\; \end{array}$$

(28) $$\begin{array}{lll} & \forall_{j}:\; & \;\;\;\;\underset{s}{\sum }x_{j,s}\leq 1\; \end{array}$$

(29) $$\begin{array}{lll} & \; & \underset{j,s}{\sum }x_{j,s}\leq \text{precs}+\text{step}\text{\_}\mathrm{size.}\; \end{array}$$

The objective function consists of three parts: protein-based and feature-based inclusion as well as exclusion. The inclusion parts contribute a positive value weighted by factors *k*_1_ and *k*_2_ while the exclusion part decreases the value of the objective function for peptide signals potentially derived from already identified proteins. It is weighted by *k*_3_. Typical values for *k*_1_, *k*_2_ and *k*_3_ are 10, 1 and 10, respectively. Constraint (25) ensures that a given protein significance is reached. It considers both the peptide probabilities of already identified peptides and the “theoretical probabilities” received from the matching probabilities of tryptic peptides and observed LC-MS features. By dividing by the transformed threshold significance *c* and the limitation *z*_*i*_≤1, additional peptide identifications of an already identified protein do not contribute to the objective function. Algorithm 1 shows the online precursor ion selection in pseudo code.

##### Algorithm 1 **Online precursor ion selection**

## Results and discussion

### Data

For evaluation of the described methods we used two samples of different complexity. On the one hand, a well-defined protein standard consisting of 5 pmol each of 48 human proteins (UPS1, Sigma Aldrich). Sample 2 is a tryptic digest of a cell lysate of HEK293 cells. It was prepared and provided by the group of Prof. H. Meyer (Medical Proteome Center, Ruhr University Bochum, Germany). The LC-MS/MS acquisition was done by Anja Resemann (Bruker Daltonics, Bremen, Germany). Both data sets were used in a previous study. See [17] for a detailed description of the sample preparation.

Peptide identification was done with X!Tandem [2] (release CYCLONE (2010.12.01)) using TOPP’s XTandemAdapter [20]. The combined target decoy version of the Swiss-Prot protein sequence database in Release 2011_08 was searched with taxonomy limited to human. Other settings included: 25 ppm mass tolerance, 0.3 Da fragment tolerance, +1 as minimal and maximal charge, methionine and tryptophane oxidation as variable modification, 1 allowed missed cleavage and tryptic cleavage sites. Additionally, carbamidomethylation of cysteines was used as fixed modification for UPS1.

We calculated peptide posterior error probabilities (PEP) using the TOPPtool IDPosteriorErrorProbability and then converted the PEPs into peptide probabilities: *p*_*i*_ = 1 - *PET*_*i*_.

For RT and detectability model training, the TOPPtools RTModel and PTModel were used. The training data sets for UPS1 consisted of three replicate LC-MS/MS runs, filtered for peptide IDs with a probability of at least 0.99 and that are part of one of the 48 constituent proteins. For the HEK293 data set, we used the same probability threshold and filtered additionally for proteins with at least 4 peptide IDs in order to keep the training sets at a reasonable size. Figure 4 shows the deviation of predicted and observed RT for HEK293.

### Evaluation workflow

**Figure 6:**
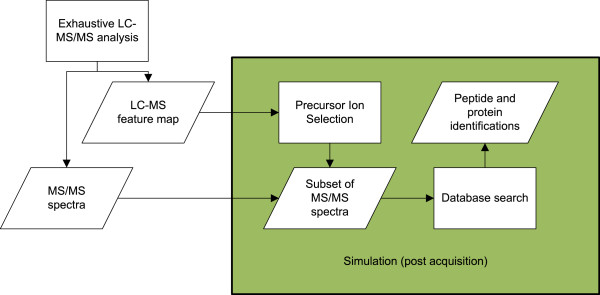
**Evaluation workflow.** First, the samples are analyzed by extensive LC-MS/MS, resulting in an LC-MS feature map and a number of MS/MS spectra. These build the data pool for all evaluation experiment that simulate precursor ion selection upon the data.

The described algorithms were tested in a variety of settings, for this reason a simulation study was best suited for our purpose. However, not the spectra themselves are simulated, only their selection (as illustrated in Figure 6). Thus, an extensive fragmentation on all possible precursors was performed. The resulting LC-MS feature map and MS/MS spectra were then used as foundation for the simulation of the precursor ion selection. This way it was possible to discriminate performance differences from differences due to technical replication. The latter pose a serious problem as was shown by Tabb et al. in a systematic study where the overlap in peptide identifications between technical replicates ranged between 35–60% [5].

### Feature-based inclusion list

The three different strategies for creating a feature-based inclusion list as presented in Figure 1 - a greedy approach (GA), data dependent acquisition (DDA), and the ILP formulation (ILP) - were evaluated with a varying maximal number of precursors per RT bin, ranging from 1 to 40. This led to inclusion lists of increasing size for each of the strategies. We used RT bins of 30 and 10 seconds for UPS1 and HEK293, respectively. Additionally, DDA in conjunction with dynamic exclusion (DEX) of each scheduled precursor for the next two fractions was included in the analysis.

**Figure 7:**
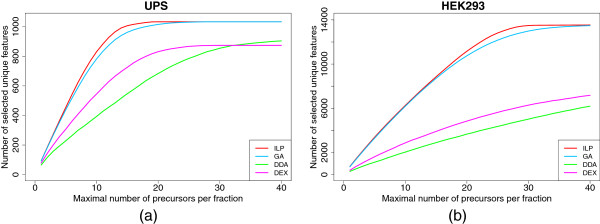
**Results of feature-based inclusion list.** For four different strategies the number of selected LC-MS features (each features counted once, even if selected several times), ILP in red, DDA in green, DEX in magenta, and GA in cyan for (**a**) the protein standard and (**b**) the complex data set.

For the evaluation, the number of uniquely selected features was counted. This means, even if a feature was selected more than once as precursor, it was only counted once. The results are shown in Figure 7. As expected, the two methods that use the feature information, ILP and GA, clearly outperform DDA and DEX. Additionally, ILP performs superior to GA as 18 precursors per RT bin are already sufficient to select all possible features as precursors. GA, in turn, needs 27 precursors per RT bin. With DDA and DEX not all features present in the dataset get selected within the limit of 40 precursors per RT bin. With the HEK293 sample both DDA and DEX perform even worse, less than half of all possible precursors are selected with a maximum of 40 precursors per RT fraction. ILP and GA perform equally well, until a maximum of 15 precursors per fraction, for higher values ILP is better. At maximal capacities of 20 and 25, which are realistic numbers within our setup, GA selects around 400 and 650 precursors less than the ILP, respectively. No method yields all 13,546 features within a limit of 40 precursors per RT bin. GA selects 13,484 features and the ILP set consists of 13,539 precursors. The advantage of our method is that it considers all features simultaneously and finds a global optimal solution. This way, more precursors can be scheduled than with a strategy that optimizes precursor ion selection for each LC-MS feature separately. 

### Protein-based inclusion list

The inclusion list creation with the protein sequence-based ILP was evaluated on the protein standard. We compared the precursors in the inclusion list with the observed features. Whenever a predicted precursor overlapped with a feature, the peptide annotation of the feature was assigned to the precursor. This way, we were able to evaluate how many peptide and protein identifications an inclusion list would deliver. This is a strong assumption as it implies that for a given feature the fragmentation works at all RT bins in a similar quality. However, as pointed out before, it is justified by the limited reproducibility of repetitive LC-MS/MS analyses.

**Figure 8:**
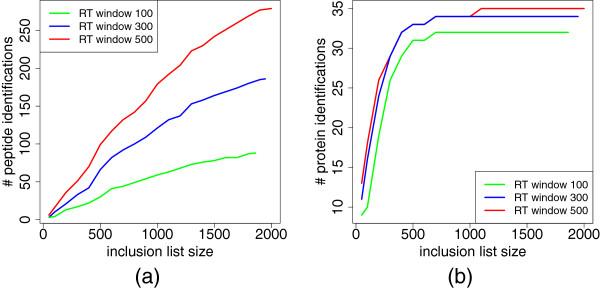
**Results of protein-based inclusion list.** Inclusion list creation via a protein-based ILP formulation for the protein standard, (**a**) the inclusion list size vs. the number of peptide identifications. (**b**) the inclusion list size vs. the number of protein identifications.

In Figure 8 (a) the number of peptide identifications for increasing inclusion list sizes is shown for RT window sizes of 100, 300 and 500 seconds. The number of identifications rises with the inclusion list size, for larger windows this effect is stronger. Their maximum width is indirectly limited by the maximum possible number of precursors per RT bin. Interestingly, when looking at the number of protein identifications, we observe a steep increase up to a size of around 500, afterwards a plateau is reached (Figure 8 (b)). Note that already around 700 predicted features yield all possible protein identifications.

**Figure 9:**
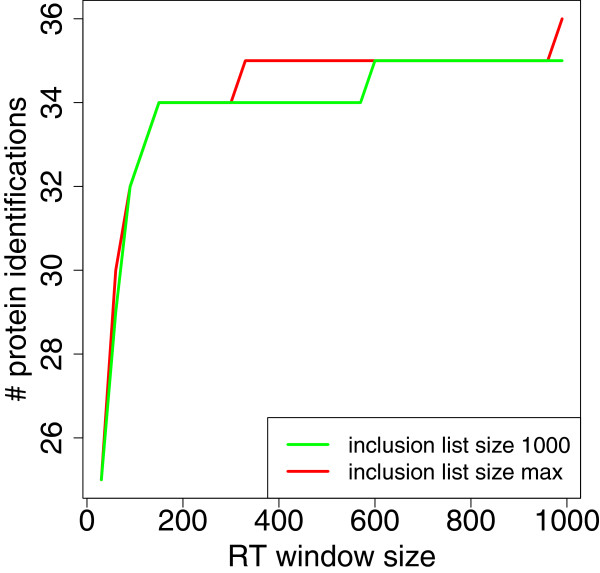
**Influence of RT window size.** Inclusion list creation via a protein-based ILP formulation for the protein standard, influence of RT window size.

We further analyzed the influence of the RT window size on the number of protein IDs. We checked sizes from 30 up to 990 seconds and using either an inclusion list with maximally 1000 entries or one of unlimited size. The results for both are similar (Figure 9). With a moderate window size of 150 seconds 34 proteins can already be identified. 

Our results show that considering the peptide-protein relation at the level of inclusion list creation prevents frequent and unnecessary identification of uninformative peptides. By incorporating protein detectabilities into the ILP we can directly control the expected protein significance. The moderate RT window sizes needed to identify a high number of proteins emphasizes further the effect of including RT predictions.

### Iterative precursor ion selection

We compared the performance of the iterative precursor ion selection with LPs (IPS_LP) with a heuristic approach presented in a previous study [17] (HIPS) and with a static precursor ion selection based on an inclusion list which is sorted by intensity (SPS).

**Figure 10:**
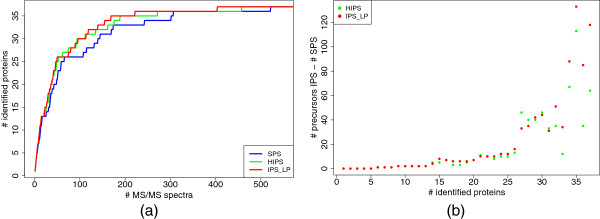
**Iterative precursor ion selection for UPS1.** Iterative precursor ion selection for UPS1 with 25 ppm mass accuracy. (**a**) shows the number of identified proteins over the number of acquired MS/MS spectra. In (**b**) the difference between IPS and SPS in the number of MS/MS spectra required for a certain number of protein IDs is shown.

Figure 10 shows the performance of the three methods applied to the UPS1 sample with a mass accuracy of 25 ppm. In Figure 10(a) the number of identified proteins over the number of selected precursors is shown. Similar to a ROC curve, a line closer to the upper left side implies a better result. All three methods start with a steep increase in protein IDs which flattens before 100 selected precursors and reaches in the end a plateau of 36 protein IDs. Finally, IPS_LP identifies the same number of proteins as the other two methods, yet by exploiting fewer precursors. This effect is shown explicitly in Figure 10(b), where the difference in the number selected precursors needed to identify a given number of proteins for the two iterative methods compared to SPS is plotted. For identifying a maximal number of proteins, IPS_LP saves a quarter of required MS/MS spectra compared to SPS. HIPS performs also better than SPS, but the advantage is smaller than with IPS_LP.

#### Sample complexity

In a previous study we observed a drawback of the heuristic IPS: it showed a clear performance loss when applied to a complex sample with limited mass accuracy [17]. This was due to erroneous assignments of theoretical peptides to observed features. In IPS_LP the specificity of assignments is increased by including RT and detectability of a peptide.

**Figure 11:**
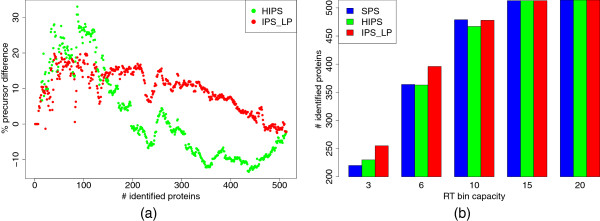
**Iterative precursor ion selection for HEK293.** Iterative precursor ion selection for HEK293 for 10 ppm mass accuracy. (**a**) shows the difference to SPS in the number of selected precursors in percent required to identify a given number of proteins. In (**b**) the total number of proteins IDs with a limited rt bin capacity is shown.

In Figure 11 the performance of HIPS is compared to SPS and IPS_LP for HEK293. For Figure 11(a), the RT bin capacity was set to at most 25. Both iterative methods require less precursors than SPS to identify up to 200 proteins. HIPS is the best method to identify up to 150 proteins as it needs up to 30% less MS/MS spectra than SPS. With ongoing analysis the advantage decreases. In order to identify 200 or more proteins, HIPS requires around 10% more precursors than SPS. On the contrary, IPS_LP is almost always better than SPS and can save a maximum of around 20% of the spectra. This shows that the heuristic makes good precursor choices at the beginning of the analysis, but after a certain number of iterations the number of false assignments of precursors to predicted peptides increases too much and thus the performance of HIPS drops. In turn, the inclusion of RT and detectability predictions increases the specificity of peptide-precursor assignments with IPS_LP.

We further examined the performance of the different methods with respect to limited number of precursors per fractions, as shown in Figure 11(b). When the maximal number of precursors in each fraction is less than ten IPS_LP is able to identify more proteins than the other methods. This implies that IPS_LP is particularily applicable in conditions with limited amounts of sample.

**Figure 12:**
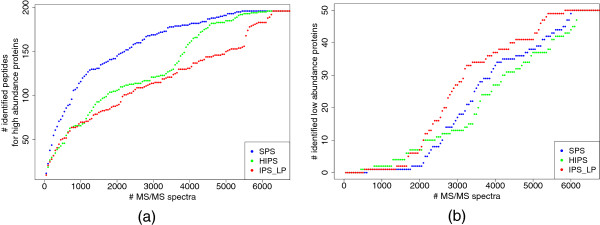
**Identification of high and low abundance proteins.** For the HEK293 sample protein abundance was estimated as the mean intensity of all features with corresponding peptide identifications. (**a**) show the number of total peptide identifications covering the 10% most abundant proteins. In (**b**) the number of identified 10% lowest abundant proteins is displayed.

As pointed out before, intensity-based selection methods like DDA identify many peptides especially for high abundance proteins. Our method aims at reducing this redundancy by excluding precursors matching peptides of already identified proteins. In Figure 12(a), we analyzed how many peptide identifications matching the 10% most abundant proteins were found with the evaluated methods. Abundance of a protein was estimated by the mean of all its corresponding feature intensities. For both IPS methods, we receive fewer peptide identifications for the high intensity proteins at the first stage of the experiment. Thus, in this part the bias towards high abundance proteins is less pronounced for IPS_LP and HIPS than with SPS. However, after 3,500 (HIPS) and 5,500 (IPS_LP) MS/MS spectra the numbers quickly reach the ones from SPS. This is due to the selection of previously downranked or excluded precursors. When considering low abundance proteins, we observe that IPS_LP identifies proteins belonging to the 10% lowest abundant ones with less MS/MS spectra than HIPS and SPS(Figure 12(b)).

#### Weights

**Figure 13:**
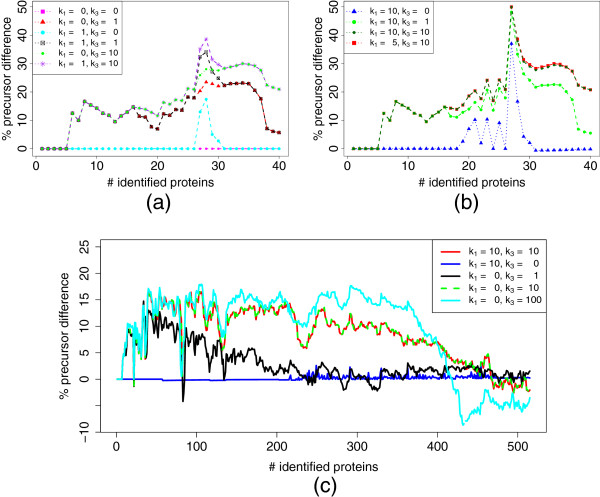
**Influence of weighting terms.** Different values for *k*_1_ and *k*_3_ were tested, *k*_2_ was fixed to 1. (**a**) and (**b**) are based on UPS1, (**c**) on HEK293.

The objective function of IPS_LP contains the weights *k*_1_, *k*_2_ and *k*_3_. We tested both samples with different values to determine how robust the system is for changes of these weights and if different samples require different weights. Both samples were examined with a mass accuracy of 10 ppm. We evaluated different values for *k*_1_ and *k*_3_ while *k*_2_ was fixed to 1 (Figure 13). Setting *k*_2_ to 0 results in early termination due to a lack of positive contributions to the objective function. As expected, *k*_1_ = *k*_3_ = 0 leads to the same performance as SPS since only feature-based inclusion is switched on. A small *k*_1_ does not greatly improve the performance as it can not compensate for the weight of all features. Especially for the complex HEK293 sample we can see that the exclusion part has a greater influence on the results. Large values for *k*_3_ lead to a good performance for a large part of the experiment, but for more than 400 identified proteins the results are worse than for SPS. This deterioration is probably due to erroneous precursor-peptide assignments and shows that too large values for *k*_3_ might impair the results. In summary, we can see that for both samples although being of different complexity the same values (*k*_1_ = 10, *k*_2_ = 1, and *k*_3_ = 10) yield good results.

#### Sequential precursor ion selection

Although with LC-MALDI MS/MS it is possible to select precursors in an order independent of their RT, in practice the sample is analyzed following a specific fraction order. Thus, in the following, we adapt the LP formulation so that it proceeds through the precursor set in a sequential order according to the fraction number.

We start with spectrum *s*^∗^ = 0. Only the capacity constraint of the LP formulation (In eq. 27) has to be adapted to account for the sequential selection:

(30) $$\begin{array}{ll} \forall_{s>s^{*}}:\underset{j}{\sum }x_{j,s} & =0\; \end{array}$$

(31) $$\begin{array}{ll} \forall_{s<s^{*}}:\underset{j}{\sum }x_{j,s} & =\text{ca}p_{s}^{*}\; \end{array}$$

(32) $$\begin{array}{ll} \underset{j}{\sum }x_{j,s^{*}} & =\text{ca}p_{s^{*}}\; \end{array}$$

Capacities of fractions with a lower number than *s*^∗^ are fixed at the number of selected precursors for the respective fraction to prevent going back in RT dimension. Capacities of fractions after *s*^∗^ are set to 0. When all precursors in *s*^∗^ were selected or its capacity has been reached, the next fraction is set as *s*^∗^.

**Figure 14:**
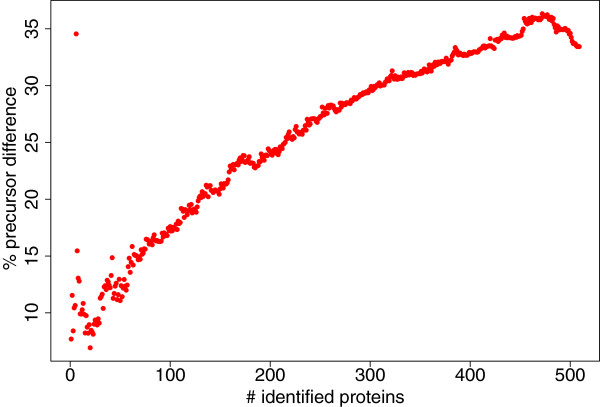
**Sequential iterative precursor ion selection for HEK293.** Iterative precursor ion selection for HEK293 for 10 ppm mass accuracy and a sequential precursor ion selection.

When selecting precursors with this sequential IPS_LP the percentage of saved precursors to reach a certain number of protein identifications rises with ongoing analysis (Figure 14). Finally, IPS_LP saves more than 30% of the precursors. As in most fractions IPS_LP selects fewer precursors than SPS, this sums up to more than 4,000 saved MS/MS spectra in the end without a loss in protein identifications. This reduction of precursors per fraction was also observed for the non-sequential IPS_LP. There, in total 7,275 were selected, in contrast to more than 13,546 with SPS.

## Conclusions

We presented methods for precursor ion selection with LC-MALDI MS/MS. We showed that inclusion list creation can be formalized as optimization problem and efficiently solved with Linear Programs. Our methods can be used to schedule an optimal set of precursors. We presented exemplarily two situations where the available information prior to MS/MS acquisition differs. When the protein sequences of interest are known our method for inclusion list creation using protein sequences is well suited, as it creates very efficient inclusion lists. Various adaptations to our methods are possible that can be easily integrated. For instance, the protein sequence-based inclusion list can be adapted to consider not all tryptic peptides of a protein but a specific predefined set of peptides that can be used for quantification of proteins in different cell states or in time series.

Finally, we presented a new method for iterative precursor ion selection that identifies proteins more efficiently than data dependent methods. This efficiency improvement is twofold: peptides from already identified proteins contribute less weight to the objective function and thus are less likely to be selected as precursors. This way the redundancy of information obtained with MS/MS can be reduced. On the other hand, IPS_LP requires considerably fewer MS/MS acquisitions to identify the same number of proteins as a static inclusion list. The remaining sample and analysis time can be used for identifying more proteins in a sample. Compared to our previously published heuristic method, IPS_LP does not suffer from massive false exclusion of signals in complex samples by incorporating machine learning methods for RT and proteotypicity predictions.

## Competing interests

The authors declare that they have no competing interests.

## Authors’ contributions

AZ developed, implemented and evaluated the described methods and wrote the manuscript. EN was involved in the experimental design. KR and HL provided supervision. KR and EN revised the manuscript. All authors read and approved the final manuscript.
